# Effectiveness of a nutrition education package in improving feeding practices, dietary adequacy and growth of infants and young children in rural Tanzania: rationale, design and methods of a cluster randomised trial

**DOI:** 10.1186/1471-2458-14-1077

**Published:** 2014-10-16

**Authors:** Kissa BM Kulwa, Roosmarijn Verstraeten, Kimberley P Bouckaert, Peter S Mamiro, Patrick W Kolsteren, Carl Lachat

**Affiliations:** Department of Food Science and Technology, Sokoine University of Agriculture, P.O. Box 3006, Chuo Kikuu, Morogoro, Tanzania; Department of Food Safety and Food Quality, Ghent University, Coupure Links 653, 9000 Ghent, Belgium; Unit of Nutrition and Child Health, Department of Public Health, Institute of Tropical Medicine, Nationalestraat 155, 2000 Antwerp, Belgium

**Keywords:** Nutrition education, Complementary feeding, Growth, Tanzania, Cluster randomised trial, Process evaluation, Intervention mapping, Theory of planned behaviour

## Abstract

**Background:**

Strategies to improve infant and young child nutrition in low- and middle- income countries need to be implemented at scale. We contextualised and packaged successful strategies into a feasible intervention for implementation in rural Tanzania. Opportunities that can optimise delivery of the intervention and encourage behaviour change include mothers willingness to modifying practices; support of family members; seasonal availability and accessibility of foods; established set-up of village peers and functioning health system. The primary objective of the study is to evaluate the effectiveness of a nutrition education package in improving feeding practices, dietary adequacy and growth as compared to routine health education.

**Methods/Design:**

A parallel cluster randomised controlled trial will be conducted in rural central Tanzania in 9 intervention and 9 control villages. The control group will receive routine health education offered monthly by health staff at health facilities. The intervention group will receive a nutrition education package in addition to the routine health education. The education package is comprised of four components: 1) education and counselling of mothers, 2) training community-based nutrition counsellors and monthly home visits, 3) sensitisation meetings with health staff and family members, and 4) supervision of community-based nutrition counsellors. The duration of the intervention is 9 months and infants will be recruited at 6 months of age. Primary outcome (linear growth as length-for-age Z-scores) and secondary outcomes (changes in weight-for-length Z-scores; mean intake of energy, fat, iron and zinc from complementary foods; proportion of children consuming 4 or more food groups and recommended number of semi-solid/soft meals and snacks per day; maternal level of knowledge and performance of recommended practices) will be assessed at baseline and ages 9, 12 and 15 months. Process evaluation will document reach, dose and fidelity of the intervention and context at 8 and 15 months.

**Discussion:**

Results of the trial will provide evidence of the effectiveness of the nutrition education package in community settings of rural Tanzania. They will provide recommendations for strengthening the nutrition component of health education in child health services.

**Trial registration:**

ClinicalTrials.gov Identifier: NCT02249754, September 25, 2014.

**Electronic supplementary material:**

The online version of this article (doi:10.1186/1471-2458-14-1077) contains supplementary material, which is available to authorized users.

## Background

Undernutrition is an underlying cause of more than one-third of global deaths in children below the age of 5 years. It is also associated with growth faltering (i.e. deficit in height or stunting), micronutrient deficiencies, delayed cognitive development and morbidity [1]. Stunting and micronutrient deficiencies are significant health problems among infants and young children in Tanzania. Prevalence of stunting among children aged 6–59 months in the national surveys of 2005 and 2009/2010 was 37.7% and 42%, respectively [2, 3]. Children in rural areas were more affected than their urban peers (40.5% vs 37.7% in 2005; 44.7% vs 32.1% in 2010). Children living in the southern and central regions of the country were more affected than those living in other regions.

Poor breastfeeding practices, inadequate dietary intake from complementary foods, high rates and repeated episodes of diseases, maternal inadequate nutritional knowledge and household food insecurity have been associated with stunting in Tanzania [4–7]. Overall, only 2 out of 10 Tanzanian children are fed in accordance with the recommended infant and young child feeding (IYCF) practices [3]. Among the breastfed children (6–23 months-old), 61% were given foods from three or more food groups, 39% were fed the minimum number of times, 30% consumed iron-rich foods and 62% consumed vitamin A-rich foods.

A formative research was conducted in one of the central regions with high rates of malnutrition to closely examine feeding practices, diets and nutritional status of infants and young children and guide decisions on improvement. The research was conducted in Dodoma in two phases. The first survey, conducted in 2009 during the post-harvest season, involved 496 households with infants aged 1 to 12 months. The second survey revisited the same households and infants in 2010 during the harvest season. Results of the surveys indicated high prevalence of stunting; 34% in 2009 and 59% in 2010. Morbidity due to acute respiratory illness (ARI) was 64% in 2009 and 57% in 2010. Diarrhoea affected 48% children in 2009 and 37% in 2010. Prevalence of anaemia was high (11 - 60% in 2009; 20 - 75% in 2010). Complementary fluids and foods were introduced at the median age of 3.0 months and 90% of children had received these foods earlier than the recommended age of 6 months. Feeding frequencies (1.7 in 2009 and 2.4 in 2010) were lower than the age-specific World Health Organisation (WHO) recommendations [8]. Portion sizes for different types of relish/sauce were small.

Similar findings in low- and middle- income countries (LMIC) have been documented in rural areas and have progressively worsened throughout the first year of life [9–13]. Systematic reviews of complementary feeding interventions have provided evidence of well adopted feeding practices, improved food recipes, increased dietary adequacy and growth and reduced prevalence of anaemia and morbidity [14–17]. The interventions included a combination of strategies, namely culturally-appropriate group nutrition education, individual counselling, interpersonal communication, home visits and mass media [18–23]. Great impact and sustainability of the interventions were achieved through participatory approaches and community involvement [24–26].

The formative research in Dodoma showed that age-specific, systematic and locally relevant interventions are needed to ensure rural children receive the best possible start in life. To address the nutrition and health situation, intervention strategies were identified and contextualised according to, among other factors, local dietary patterns, meal preparation, food beliefs and preferences, food availability and cost and ability to change attitudes. The strategies were packaged as a nutrition education package. Opportunities that could optimise successful delivery of the package and encourage behaviour change include willingness to modifying some feeding options, seasonal availability and accessibility of foods, established set-up of village peers and existence of health centres and health staff in some villages. This paper describes the rationale, design and methods of a cluster randomised controlled trial to evaluate the effectiveness of a nutrition package in rural Tanzania.

### Objectives and hypothesis

The primary objective of the intervention trial is to implement and evaluate the effectiveness of a nutrition education package in improving infant and young child feeding practices, dietary adequacy and growth. The trial hypothesises that the nutrition education package will be more effective than the routine health education. The primary outcome will be linear growth as assessed by change in length-for-age Z scores. Secondary outcomes will include feeding practices, nutrients intake and level of knowledge on recommended practices. Results of the trial are expected to provide evidence to the effectiveness of the package in community settings and, where appropriate, recommendations to strengthen the nutrition component of health education in child health services.

## Methods/Design

### Development of the nutrition education package

Intervention Mapping and Theory of Planned Behaviour [27, 28] provided the theoretical framework for the design and development of the package and the intervention. The intervention mapping framework integrates theory, empirical findings from the literature and information collected from the target population to develop culturally appropriate and theoretically sound interventions [29, 30]. The framework has been used successfully to develop health behaviour interventions [31, 32]. This study applied consecutively and iteratively the six steps of intervention mapping.

#### Step 1: Needs assessment

Needs assessment involved a literature study on complementary feeding strategies in LMIC, review of secondary data on child health and the formative research conducted in 2009 and 2010 in the study area. Key informant interviews, focus group discussions, food market surveys and household surveys were conducted to obtain in-depth insight into the nutrition and health situation of infants and young children, community and household factors, parents’ motivations and behaviours that facilitate and constrain infant and young child feeding and health.

#### Step 2: Identifying intervention performance and change objectives

Results from *Step 1* were used to identify behaviours, motivations, barriers, predisposing and enabling factors associated with inadequate dietary intake, morbidity and poor growth. A conceptual framework illustrating expected behaviours and mediating factors for improvement of child growth was developed (See Additional file 1). Aspects that will be implemented by the intervention are also presented. The overall intervention objective “improve feeding practices, dietary adequacy and growth of infants and young children (6–15 months)” was formulated and divided into sub-objectives and behavioural performance objectives (i.e. target behaviours that have to be accomplished by the target group to achieve the intervention objective). A matrix of intervention objectives and behavioural performance objectives, classifying individual and environmental determinants as mediating mechanisms and modifying conditions was developed (See Additional file 2). Discussions with district health experts and village health committees helped to refine the intervention further.

#### Step 3: Selection of theory-based intervention methods and techniques

Theoretical methods considered to influence changes of performance objectives (i.e. target behaviours) were identified and practical strategies were selected to put the theoretical methods into practice [31]. Systematic reviews [14–16], individual nutrition education and counselling interventions [20, 21] and summary of theoretical methods [31] were used in the selection of existing methods and techniques, and the development of new strategies which fitted our intervention and behavioural objectives. Theory of Planned Behaviour was used as the main focus of behavioural change during the selection of theory-based methods and strategies [33]. The theory has been widely employed in health and nutrition behaviour change studies [28, 34, 35], including infant feeding [36–38]. The theory facilitates an understanding of how behavioural intentions influence actual behaviour change, with skills, abilities and environmental factors acting as important moderators of change.

The theoretical methods were translated into practical strategies and these were related to the levels of the intervention. An example includes a behavioural objective and outcome expectation of “adding mashed animal-source food to infant meals at least 3 times per week would provide nutrients to make a child grow well and offer protection against common illnesses.” A mother will be willing and more likely to apply the recommendation using the following methods and techniques:

- the individualisation method to provide opportunities for mothers to have personal questions and concerns about child feeding answered (e.g. what to feed, how to feed) or instructions paced according to individual progress;
- cognitive skills training with guided practice to influence self-efficacy;
- tailoring to increase practical knowledge on selection and preparation of new food recipes; and
- mobilising social support (e.g. trained peers, sensitised family members and health facility staff) to offer encouragement and support, and likely to help the mother to maintain optimal child feeding practices.

#### Step 4: Developing the intervention and pre-testing

The intervention objectives, selected methods and strategies were combined and a nutrition education package was developed. The nutrition education package is composed of four components, namely 1) education and counselling of mothers, 2) training community-based nutrition counsellors and monthly home visits, 3) sensitisation meetings, and 4) supervision of community-based nutrition counsellors. Draft versions of the package were discussed with district health and nutrition experts, village health committees and fine-tuned by the research team. Behavioural change at the individual level is intended for mothers of rural infants aged 6 months through education and counselling sessions and cooking demonstrations. Based on local typical meals, the intervention will promote preparation and consumption of a variety of foods. Behavioural change at the family level will consist of sensitisation meetings with family members of targeted infants. This will help to create a nutrition-promoting environment. At the community level, the intervention will train community-based peers such as village health workers, lay counsellors and resource persons to support mothers and families through home visits. In addition, sensitisation meetings with health facility staff will help to reinforce and support mothers’ behaviour change.

Training and educational resources were developed to support the implementation of the package. These include: 1) Training guide for community-based nutrition counsellors, 2) Manual for community-based nutrition counsellors, 3) Educational plan for mothers, 4) Information booklet for mothers and families, and 5) Counselling cards. The resources contain topics on breast feeding, complementary feeding, selection and preparation of locally available foods, feeding during illness, healthcare-seeking, hygiene and home environment sanitation. In addition to these, resources for nutrition counsellors cover communication skills, behaviour change communication and counselling skills. The developed resources were pre-tested with a convenient sample to assess whether the content and format were realistic, understandable, culturally appropriate, visually appealing and motivating.

#### Step 5: Implementation plan of intervention

The intervention strategy is centred on the child with their mothers as the primary target group. To stimulate lasting behaviour change, ensure continuity, and enhance linkage between communities and health facilities, secondary target groups have been incorporated in the intervention. These include family members (fathers, grandmothers, school-going siblings), community-based nutrition counsellors and healthy facility staff. Supportive structures such as district health management team and village health committees will be consulted regularly to enhance intervention support and feedback.

#### Step 6: Evaluation plan of intervention

The effectiveness of the nutrition education package is evaluated in a cluster randomised controlled trial, whereby villages will be randomly allocated to receive a nutrition education package or routine health education. The intervention group will receive the nutrition education package, whereas the control group will continue to receive routine health education given monthly at health facilities. Children aged 6 months and their families will be recruited and followed up for 9 months. Extensive process evaluation will also be performed to document reach, dose and fidelity of the intervention trial.

### Study setting

The intervention will be conducted in Mpwapwa District in Dodoma Region, central Tanzania. Dodoma is among the regions in the country with the highest prevalence of stunting, 44.4% in 2005 and 56.0% in 2010 [2, 3]. The District has a total population of 304,096 and 67,577 households [39]. The region is predominantly semi-arid, characterised by a short single wet season (November to March) and a long dry season (May to mid-November). Subsistence farming and traditional rearing of animals are the primary economic activities. The district health system makes child health services available through a network of 43 dispensaries, 2 health centres and 1 district hospital. Mpwapwa district is administratively divided into 3 divisions, 18 wards, and 84 villages [39]. A village is the lowest administrative unit and is considered as a geopolitical unit and intact social group. It has a local government authority and committees. Each village usually has two village health workers. They work as volunteers, mainly to assist health facility staff during immunisation and child growth monitoring activities.

### Study design

**Figure 1:**
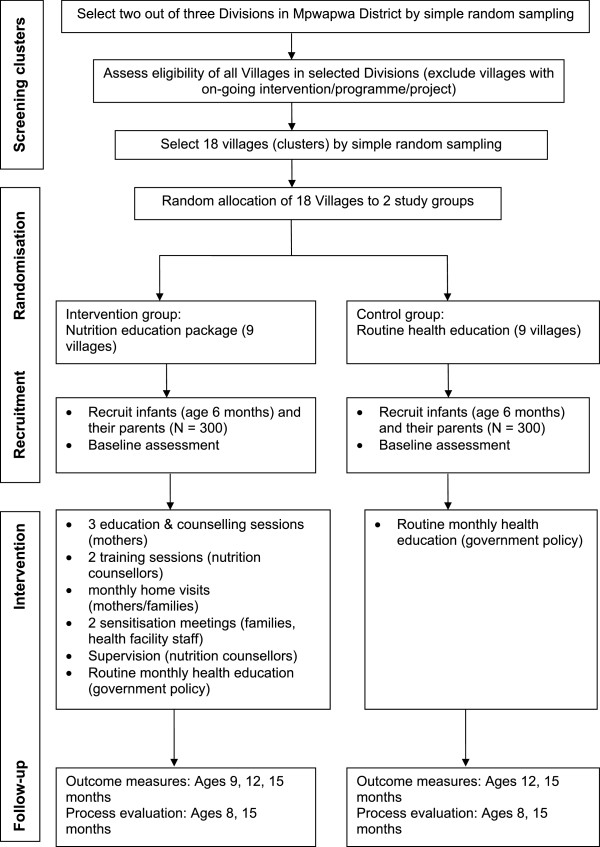
**Trial profile.**

The intervention trial will use a parallel cluster-randomised controlled trial design (Figure 1) and will be conducted and reported in line with the CONSORT recommendations for cluster randomised trials [40]. Because the intervention will be delivered in community settings and encourage collective participation at household settings, to minimise contamination and facilitate logistical convenience in delivery, the unit of randomisation will be the village. Villages will be randomly assigned to either the intervention or control study groups. The intervention duration will be 9 months. Infants will be recruited when aged 6 months and followed up until they attain 15 months.

The control group will continue to receive routine health education, a standard government health service for children below the age of five years, offered monthly by health staff at health facilities. Routine health education offers education to mothers during growth monitoring and immunisation contacts. The sessions are usually short (10–15 minutes) and focus on general health issues including child feeding, prevention of diseases such as malaria and importance of immunisations.

The intervention group will receive the nutrition education package in addition to the routine health education. Mothers and caregivers will receive 3 sessions of education and counselling on optimal infant and young child feeding and health, when the child reaches 6 months, 9 and 12 months. Nutrition counsellors will receive training at baseline and the session will be repeated after 6 months. After training, the nutrition counsellors will conduct monthly home visits to counsel and support mothers and their families. Supervisory visits will be conducted by the research team after every 2 months to assess counsellors’ work. Separate sensitisation meetings with families and health staff responsible for child health will be conducted at baseline and meeting will be repeated after 6 months.

### Sampling and randomisation

A village forms a unit of randomisation for the trial, while infants and their parents within the village will form units of observation. Two out of three Divisions (Kibakwe, Mpwapwa, Rudi) in the district will be selected by simple random sampling. A list of all villages in the selected divisions will be compiled, and villages found to have an on-going intervention or project will be excluded. Eighteen villages will be selected from the list by simple random sampling. The preselected villages will be listed alphabetically. A simple randomisation with a 1:1 allocation will be used to randomise villages to either control or intervention group. A list of random numbers will be generated in MS Excel 2007 and the generated values will be fixed by copying them as “values” next to the alphabetic list of the preselected villages. These will then be arranged in ascending order according to the generated random numbers: and the first 9 will be selected as intervention clusters and the last 9 as control clusters.

Generation of allocation sequence and randomisation of clusters will be done by a statistician blinded to study groups and not participating in the research. Data collectors will be masked of the village allocation by not informing them of the allocation, not being part of trial implementers and not being residents in any of the villages. However, trial participants may know if they are in the intervention or control group.

### Sample size estimate

The sample size was calculated for unmatched cluster-randomised trials [41] as follows:


whereby: **c** is the number of clusters required in each treatment group; **Z**_**α/2**_ is the standard normal distribution corresponding to the 95% degree of confidence on a two-sided test; **Z**_**β**_ is the standard normal distribution corresponding to 80% power; **σ**_**0**_ and **σ**_**1**_ are the within cluster standard deviations of the outcome variable in the presence and absence of intervention, respectively; **n** is the minimum number of individuals sampled in each cluster; **k** is the coefficient of variation of true means between clusters within each group; and **μ**_**0**_ and **μ**_**1**_ are the within cluster population means in the presence and absence of intervention, respectively.

As there is no information available on the intra-cluster coefficient of variation, the estimate for the between-cluster coefficient of variation (k) was based on conservative estimates of ≤0.25 and 0.15-0.25 experienced in similar health and nutrition studies [41, 42]. A coefficient of variation between clusters (k) of 0.1 was assumed and 30 infants per cluster based on district’s estimates of birth rates. Data from the 2009 formative survey were used to make assumptions on change of values of the primary outcome length-for-age Z-scores (LAZ): a mean LAZ of −1.5 and a standard deviation of 1.3 in the control group. From the formula, 8.9 (~9) clusters will be needed to detect at least a 0.35 difference in LAZ between intervention and control groups, with the power of 80% (Z_β_ = 0.84) and a statistical significance of 5% (p < 0.05; Z_α/2_ = 1.96, two-tailed test). A total of 270 infants will be enrolled in each study group. Allowing for a 10% loss to follow-up, a sample size of 297 infants, i.e. 33 per village, will be required. A total of 594 (~600) infants will be recruited.

### Recruitment and collection of baseline information

After cluster randomisation, sub-village leaders will identify all infants aged 6 months and their parents in a systematic door-to-door survey. Parents of identified infants will be invited to a meeting where the nature and purpose of the trial and eligibility criteria will be explained. Thereafter, the infants will be screened for eligibility. **Inclusion criteria** will be breastfeeding infants aged 6 months; parents (or caregivers) are residents in the sampled village and have no plans to move away during the intervention period. **Exclusion criteria** will be infants with obvious congenital or chronic abnormalities impairing feeding or physical growth measurements; have oedema; are severely ill or have clinical complications warranting hospitalisation. Parents/guardians and infants who meet the inclusion criteria, agree to participate in the trial and give a written informed consent will have their infants recruited in the trial. Baseline information of recruited infants and their parents/guardians will be collected using a structured questionnaire. The information will include household’s, parents’ and infant’s characteristics. Structured interviews with village and sub-village leaders will also be conducted to collect village information (i.e. population, health facility, water supply, schools, livelihoods, resources, economic opportunities).

### Ethical considerations and permissions

The trial will be explained in a formal letter and verbally to district administrative and health officials. Approval to include the selected villages in the trial will be sought from village authorities. After identification of eligible infants in each cluster, their parents/guardians will be invited to a meeting where nature and purpose of the trial will be explained. The information sheet and informed consent document will be tailored to the study group (intervention or control) to which a particular cluster was allocated [43]. Parents/guardians who agree to participate in the trial will be requested to sign a written informed consent. A verbal consent will be requested from parents or guardians who are unable to read and/or write. An impartial witness will be present when the verbal consent is given. Informed consent will also be sought from nutrition counsellors and health facility workers in intervention villages. During the course of the trial, infants reporting to be ill in both study groups will be referred to health facilities where they usually receive treatment free of charge according to Ministry of Health regulations. Households that do not participate in the intervention group will receive education and counselling resources at the end of intervention. Ethical clearance was obtained from the National Institute of Medical Research (Tanzania), Institute of Tropical Medicine (Belgium) and University of Antwerp (Belgium).

### The intervention

The language of communication during the intervention delivery will be Kiswahili, a written and spoken language in the country.

### Component 1: Education and counselling of mothers

This component aims to impart rural mothers and caregivers with action-oriented knowledge, skills, behaviour and attitude that will motivate their ability to adopt optimal infant and young child feeding and health care practices. Nutrition education and counselling will be conducted with mothers of recruited infants in three sessions. The first session will be conducted when infants are 6 months-old. The second and third sessions will be conducted when infants are aged 9 and 12 months, respectively. Each session is expected to last 2 to 3 hours. The content is consistent with age-specific feeding recommendations made by WHO [8, 44] and timed to coincide with child’s age at 6, 9 and 12 months. The sessions will be centred on feeding, health, hygiene and sanitation. Distribution of sessions and topic coverage is presented in Table 1. Theoretical sessions (Part 1) will be followed by cooking demonstrations (Part 2) and counselling for mothers who have individual concerns (Part 3).

**Table 1:** **Education and counselling sessions for mothers when children attain different ages**

Session 1	Session 2	Session 3
Child 6 months-old	Child 9 months-old	Child 12 months-old
**Part 1:** Group education	Feedback and review of previously set goals	Feedback and review of previously set goals
	**Part 1:** Group education	**Part 1:** Group education
Breastfeeding your baby	Breastfeeding your baby	Breastfeeding your baby
Feeding your baby from 6 to 8 months (diversity, frequency, consistency, amount, responsive style)	Feeding your baby from 9 to 11 months (diversity, frequency, consistency, amount, responsive style)	Feeding your baby from 12 to 23 months (diversity, frequency, consistency, amount, responsive style)
Locally available foods for preparation of nutritious meals	Locally available foods for preparation of nutritious meals	Locally available foods for preparation of nutritious meals
Feeding your baby during and after illness	Feeding your baby during and after illness	Feeding your baby during and after illness
Hygiene (safety, handling & storage of food) and Home environment sanitation	Hygiene (safety, handling & storage of food) and Home environment sanitation	Hygiene (safety, handling & storage of food) and Home environment sanitation
Health care-seeking and Disease prevention	Health care-seeking and Disease prevention	Health care-seeking and Disease prevention
Utilisation of child health services and compliance to treatment and advice	Utilisation of child health services and compliance to treatment and advice	Utilisation of child health services and compliance to treatment and advice
**Part 2:** Cooking demonstration	**Part 2:** Cooking demonstration	**Part 2:** Cooking demonstration
**Part 3:** Individual counselling	**Part 3:** Individual counselling	**Part 3:** Individual counselling

Direct (teacher-directed), interactive (discussion, sharing) and experiential (learner-centred, activity-oriented) instructional strategies will guide the approach of delivering education sessions. Talks, group discussions, group work exercises, demonstrations, role plays, storytelling, simulation, case studies and problem solving will be used to impart knowledge and skills, enhance maternal attitude and self-efficacy. For example, the topic “Locally available foods for preparation of nutritious meals” will apply the following teaching methodologies:

i. a lecture/talk will explain about nutrients in foods and their role in promoting growth and good health, and mothers will role play nutritional benefits;
ii. group work will involve mothers in identification and grouping of locally available foods for making typical household meals;
iii. mothers will be presented with case studies of meals to evaluate inadequacies, discuss and apply problem solving skills to overcome the inadequacies;
iv. key recommendations (Table 2) for inclusion of varieties of foods to increase content and intake of nutrient density of meals will be summarised in a lecture and mothers encouraged to make implementation goals; and
v. cooking demonstrations will show how food varieties can be used to prepare nutritious meals.

**Table 2:** **Key recommendations and messages for promoting optimal feeding and health practices**

1	Continue to breastfeed your child on demand, during the day and night throughout the first and second years. Breastfeed first before giving other meals.
2	Start giving soft and thick meals in addition to breast milk when child completes 6 months, then continue with mashed and semi-solid meals. Increase variety, amount and consistency of food as he/she becomes used to eating and chewing different foods.
3	Prepare a thick porridge made from a combination of cereal flours. Enrich the porridge by adding groundnuts, milk, egg or legume. Thin porridge does not contain adequate nutrients to support your baby to grow well and stay healthy.
4	Give undiluted cow’s milk to your child at least 3 times per week.
5	Cook, mash, and add one or more ingredients from legumes (e.g. beans, pigeon peas, bambara groundnuts, cowpeas) in each meal.
6	Cook, mash and feed animal source foods (e.g. eggs, beef, pork, chicken, liver, fish, sardines) at least 3 times per week.
7	Cook, mash, and feed vegetables (e.g. leafy vegetables, pumpkin, avocado) in each meal.
8	Feed your child a fruit (e.g. pawpaw, ripe banana, mango, orange) after a meal at least once per day.
9	Increase frequency of feeding meals per day (2–3 times at 6–8 months, 3–4 times at 9–11 months, 3–4 times at 12–23 months). Feed 1–2 snacks (e.g. fruit, bread with groundnut paste) between two major meals.
10	Encourage your child to eat with patience and love. Encourage a sick child to drink and eat more frequently during illness. Provide extra food after illness to facilitate quick recovery.
11	Wash your hands with soap before preparing meals or before feeding children. Wash your hands with soap after visiting the toilet, and after cleaning a child who has defaecated.
12	Learn to recognise early danger signs and symptoms of childhood diseases. Promptly take a sick child to the nearest health facility for examination and treatment.
13	Ensure that your child completes a full course of prescribed medications. Return for a follow-up visit at the health facility when required or if child’s condition is not improving.
14	Keep your home environment clean. Use preventive measures to protect your child and family from diseases.

Cooking demonstrations aim to show how different food items can be used to prepare nutritious meals, illustrate appropriate amount and consistency of meals and demonstrate responsive feeding. Procedures for preparing nutritious meals based on promoted recipes will be described during the sessions. The recipes are based on meat, legumes, vegetables and root/tuber, and the foods can be eaten alone as a complete meal or eaten with cooked staple (stiff porridge *ugali*, rice, green banana, cassava). Under the guidance of the researcher, mothers of recruited infants and community-based nutrition counsellors will centrally prepare meals based on proposed recipes. One portion will be fed on-site and another given for feeding at home on the same day. Responsive feeding styles will be practiced by mothers during the feeding sessions and observed. For each cooking session, recipes will be alternated to reduce monotony, improve texture and flavour, and stimulate reflex chewing. Mothers will be encouraged to try the recipes and other feeding recommendations at home. Information booklets containing topic content, recipe examples and promoted recommendations will be distributed after the first session.

After cooking demonstrations, mothers will be invited for individual counselling. The counselling sessions will include listening, learning about their difficulties and assessing the situation, providing relevant information, and building mother’s confidence to modify behaviours and overcome barriers. The mother will be referred to nutrition counsellors in her village for support and follow-up during the monthly home visits. Follow-up visits at home will be done by community-based nutrition counsellors. After completion of each session (Part 1, 2, and 3), mothers will be encouraged to set achievable goals (i.e. to prompt intention to try the recommendations). Feedback regarding the set goals will be reviewed during the next education session.

### Component 2: Training community-based nutrition counsellors and monthly home visits

This component aims to empower community-based nutrition counsellors with action-oriented knowledge and skills to effectively counsel and negotiate with mothers and families to adopt recommended practices. In each intervention village, 2 individuals will be recruited and trained to carry out four major tasks: 1) counsel and support mothers and their families during home visits, 2) refer infants identified with danger signs of illnesses and malnutrition to health facilities, 3) supervise cooking demonstrations during the education sessions and 4) mobilise mothers for outcome assessments. Potential individuals will be nominated by village leaders in their respective villages, and the research team will interview and recruit the most suitable candidates. To be selected, a candidate has to be aged between 25 and 45 years, be a resident in the village with no plans of leaving within a year, has completed at least 7 years of formal education and has personal experience in infant feeding. In addition, the candidate will have to demonstrate that they have time, are eager to participate in the trial and willing to undertake home visits.

All recruited nutrition counsellors will be centrally trained for 3 days. The first training session will be conducted at the beginning of the intervention whereas the second session of similar content will be repeated 6 months later (i.e. when infants are aged 12 months). The training will contain three modules. **Module 1** will focus on orientation and introduction, why nutrition matters during the first two years of life, feeding principles and practices, use of locally available foods to make nutritious complementary meals, child health and preventive measures. **Module 2** will focus on communication skills, behaviour change communication, counselling skills and negotiation, problem solving (trying different solutions to common problems), use of counselling resources, field practice and feedback on field practice. **Module 3** will focus on home visits tasks and procedures, use of counselling resources, supervisory visits and training evaluation. A standardised test with multiple and open-ended questions will be given before and after training to test knowledge and attitudes about child feeding and health. The training methodology will include lectures, small group activities and discussions, plenary discussions, demonstrations, role plays, and hands-on field practice with mothers in villages which will not be part of the intervention. Theoretical training will be followed by practical exercises designed to test performance. During the practice sessions, they will be observed and their skills discussed for improvement during plenary sessions. After training, counsellors will be issued counselling resources (counselling cards, workbook, family card, referral slip, counselling manual) as home visiting tools.

The nutrition counsellors will begin home visits immediately after their training. Each nutrition counsellor will be assigned 10–12 mothers residing in their village and each mother will be individually visited and counselled. They will make home visits once per month for 9 months of the intervention period. Mothers with sick children or children with feeding problems will be offered additional visits. The counselling will take place at home to ensure other family members can also take part in the sessions. The counsellors will be responsible for keeping home visits records in workbooks. These will include:

- behaviour adopted by mothers;
- issues discussed, barriers, next/missed appointments;
- referrals to health facilities given to infants with danger signs of childhood illnesses; and
- in-depth information of best-case and worst-case household circumstances for lessons learned during the intervention.

Achievements and unresolved difficulties during the visits will be discussed during supervisory or monitoring visits.

### Component 3: Supervision of community-based nutrition counsellors

The aim of supervising nutrition counsellors is two-fold: 1) to reinforce theoretical knowledge, counselling and practical skills learned during training, and 2) to give support and feedback needed to address child nutrition challenges in their communities. Supervisory visits coupled with observed counselling sessions, review of home visits workbooks and discussions on feedback will be made by the research team to assess the quality of counsellors’ work. An observation checklist for individual counselling will be used to assess practice on interpersonal skills, active listening, effective use of counselling resources, offer referrals appropriately, promote problem-solving among mothers, and discuss practical solutions. Counsellors will receive a simple score of 2 = “Yes, sufficient” or 1 = “Yes, limited” if a correct practice is observed, and 0 = “None at all” if the practice is not done. Supervisory visits will take place in the villages at 2-weeks interval in the first 2 months of intervention, and thereafter visits will be made at 2-month interval.

### Component 4: Sensitisation meetings

Sensitisation meetings will be held in intervention villages in the form of presentations and discussions with selected groups likely to influence the success and sustainability of the intervention. These include family members and health facility staff.

#### Sensitising family members

The aim of sensitising family members is to improve their knowledge and attitude on child nutrition issues so as they can ensure dietary adequacy and optimal growth. A 1 to 2 hour sensitisation meeting will be held with fathers, grandmothers and older siblings of recruited infants. The structure of the meeting will consist of four parts: Intervention rationale, content and activities; why nutrition matters during the first two years of life; promoted recommendations and their role in supporting mothers; and importance of adherence to intervention for its success. The meetings will take place twice: at baseline and 6 months into the intervention.

#### Sensitising health facility staff

The aim of sensitising health facility staff is to improve their knowledge and attitude on feeding practices so as they can counsel mothers and caregivers during routine well-baby and sick-child visits. In villages where a health facility exists, two potential health workers will be nominated by their resident in-charge of the health facility to participate in sensitisation meetings. A 3 to 4 hour sensitisation meeting will take place in respective villages. At the meeting, the intervention will be outlined and its implications for community infants and young child discussed. Other topics for presentation and discussion will include why nutrition matters during the first two years of life; feeding principles and practices, use of locally available foods to make nutritious complementary meals, and key messages to share with the mothers. The meetings will take place twice: at baseline and 6 months into the intervention.

### Data collection and measuring impact of the intervention package

Procedures for the collection of information are standardised. Data collection forms (structured questionnaires, observation checklists, etc.) were translated into the local language, Kiswahili. The accuracy of the translation was checked by back translation into English. The questionnaires were pre-tested with 20 mothers in a neighbouring district to determine whether the items were adequate, clear and culturally appropriate. Revisions and modifications were applied accordingly. Data collectors will administer the forms in Kiswahili. After data collection, all filled forms will be manually checked for completeness and consistency. To enhance blinding, precise objectives of the study and village allocation to trial will not be disclosed to data collectors, nutrition counsellors will not be responsible for data collection, and data collection schedule will be randomised.

### Primary outcome

The primary outcome of the study will be linear growth as assessed by mean change in LAZ at 6–12 and 6–15 months of age. Data will be collected at baseline, and ages 9, 12 and 15 months in the intervention group, whereas data will be collected at baseline, and ages 12 and 15 months in the control group.

### Secondary outcomes

Secondary outcomes will include:

- Mean change in weight and weight-for-length Z-scores (WLZ)
- Mean intake of energy, fat, iron and zinc from complementary foods
- Proportion of children consuming foods from 4 or more food groups
- Proportion of children consuming the recommended number of semi-solid/soft meals and snacks per day
- Maternal level of knowledge and practice of recommended feeding and health practices

#### Anthropometry

Recumbent length will be measured to the nearest 0.1 cm using a portable wooden infant/child length board (Shorr Productions, Perspective Enterprises, Portage, Missouri) with a fixed head and sliding foot piece. Children will be weighed with light clothing to the nearest 10 g using a digital infant weighing scale (Salter model 235, CMS Weighing Equipment, London). The weighing scale and length board will be placed on a flat surface to ensure correct measurements. Each measurement will be done in duplicate and the mean value calculated. Child’s age will be recorded from the health card or birth certificate. Standardised anthropometric procedures [45] will be observed. Nutritional status indices LAZ and WLZ will be computed for each child by comparing the child’s measurements with the reference values of the WHO 2006 child growth standards using ANTHRO software v3.0.1 (ANTHRO, WHO, Geneva). Data will be collected at baseline, 9, 12 and 15 months in intervention group, whereas in control group, data will be collected at baseline, 12 and 15 months.

#### Dietary intake

Intake of specific nutrients (energy, protein, fat, iron, zinc) will be assessed at baseline and 12 months in intervention and control groups using interactive 24-hour dietary recall [46]. Mothers will be asked to recall all foods and fluids consumed by their children in the previous 24 hours. Information on time, type of meal, ingredients used, amount of total dish, and amount consumed will be documented. Amounts will be determined using a digital food balance (TANITA kitchen scale, KD-400, maximum weight 5 kg, precision 1 g), household and other common utensils (e.g. spoons, cups, bowls), and visual aids (e.g. fresh foods). Daily intake of nutrients will be calculated using the 2008 Tanzania Food Composition Tables [47]. Nutrients intake will be compared to international recommendations [48, 49].

#### Dietary diversity assessment

Information from the interactive 24-hour food recall will be used to generate number of food groups consumed by children at baseline and 12 months. At 9 and 15 months, a structured questionnaire will be used to document type of meals consumed by children in the previous 24 hours. Meals of each child will be separated into their ingredients and the number of food groups consumed generated as recommended by Dewey and colleagues [50]. The food groups includes: (1) cereals, tubers and roots; (2) legumes, nuts and seeds; (3) milk and milk products; (4) meat, fish, poultry, liver/organ meats; (5) eggs; (6) vitamin A-rich fruits and vegetables; (7) other fruits and vegetables; and (8) oils and fats. Sum of total number of food groups consumed will be generated for each child. Proportion of children consuming meals from 4 or more food groups will be determined at ages 9, 12 and 15 months in intervention villages and at 12 and 15 months in control villages.

#### Feeding frequency

Number of semi-solid/soft meals and snacks consumed per day will be collected using a structured questionnaire. Proportion of children consuming the recommended number of semi-solid/soft meals and snacks per day according to WHO [8, 51] will be determined at ages 9, 12, and 15 months in intervention villages and at 12 and 15 months in control villages.

#### Maternal knowledge of recommended practices

The intervention will promote adoption of key feeding and health care recommendations during the education and counselling sessions and home visits. Maternal recall of key recommendations will be assessed using a list of structured and open-ended questions generated from the list of key recommendations. Spontaneous recall questions will be used (e.g. What signs of illness would indicate that your child was very sick and is in need of urgent attention/treatment?), as well as prompted recall (e.g. Have you heard anything about children requiring more food and fluids during illness? This will be followed by a query of messages associated with it. Mothers will receive a simple score of 1 for each correct response and 0 for wrong response.

#### Maternal practice of key recommendations

Maternal practice of key recommendations will be assessed using a structured questionnaire and structured observation. Direct questions based on education lessons and the 11-item key recommendations will be asked to mothers. For example, a recommendation to “Wash your hands with soap before preparing meals or before feeding children,” mothers will be asked to name occasions when they wash their hands with soap, why they should wash their hands, and how they wash their hands. For recommendations on consumption of specific foods (e.g. undiluted cow’s milk, legumes, etc.), a 7-day food frequency questionnaire will be used to determine consumption frequency of the specified food items. The list of such food items will be read to mothers and asked whether they had fed those items in the previous 7 days. Pre-coded categories will include never, once per week, 2–4 times per week, 5–6 times per week, once per day, 2–3 times per day, 4 or more times per day [52].

Information on maternal recall and practice of key recommendations will be collected in intervention villages at ages 9, 12 and 15 months.

### Other measurements

Information on factors that may influence the effectiveness of intervention is collected at baseline in both intervention and control groups. A structured questionnaire will be used to collect household, parents (or caregivers) and child characteristics, and other factors that may confound outcomes:

- Household: headship, composition, housing, fuel for cooking and lighting, water availability, and sanitation
- Maternal and paternal: age, education, marital status, occupation and level of child support
- Child: birth order, birth weight, early infant feeding [breast feeding, exclusive breast feeding, age at first introduction (and types) of fluids/foods], child health [place of delivery, past immunisations, morbidity and healthcare seeking, haemoglobin concentration]
- Child health during the intervention period
  - ○ *Immunisation status* will be verified using a child’s health card at 9 months.
  - ○ *Child morbidity and healthcare-seeking practices* within two weeks before the visit will be assessed through maternal recall of signs and symptoms related to acute respiratory infections, diarrhoea, and fever; duration and severity; treatment sought; prompt use of medication; place of treatment; type of transportation used to reach the health facility; reasons for not attending a health facility; mother’s ability to identify danger signs of childhood illness; and mother’s perceptions of appropriate care after being presented with scenarios of sick children with danger signs. Acute respiratory infections will be defined as the presence of mucopurulent nasal discharge with or without cough, difficulty breathing, fast breathing than usual, breathing with severe noise or wheezing or difficulty inhaling. Diarrhoea will be defined as three or more liquid or semi-liquid stools in 24 hours. Fever will be based on maternal report of elevation of infant’s body temperature above normal. Morbidity and healthcare seeking data will be collected at baseline and at ages 9, 12, and 15 months in intervention group, whereas the same information will be collected at baseline and at age 12 months in the control group.
  - ○ *Haemoglobin concentration* will be determined using a portable battery-operated electronic Haemoglobin Photometer (HemoCue Hb 201+, Angelhom, Sweden). Non-fasting blood samples will be obtained from a finger prick of each child between 08.00 and 10.00 hours using sterile butterfly lancets. A drop of blood will be placed in a sterile microcuvette and inserted in the machine for immediate reading. The machine will be calibrated daily with a reference microcuvette provided with the machine. A trained and skilled clinician working at the Mpwapwa District hospital will be responsible for collection of blood samples and haemoglobin determination. Haemoglobin concentration will be assessed at baseline and age 12 months in both intervention and control groups. Children found to be anaemic (haemoglobin concentration below 11.0 g/dl) during the assessments will be referred to the nearest health facility for treatment free of charge, and later, closely followed up to monitor compliance to treatment and advice.
- Seasonality: pre-harvest, harvest, post-harvest and rainy or dry season

### Process evaluation

A process evaluation will be conducted to document the intervention implementation process so as to 1) assess whether the intervention activities are implemented as planned (i.e. fidelity), 2) evaluate the extent to which the intervention reaches the intended mothers and their families (i.e. reach), 3) determine the degree to which targeted mothers are exposed to intervention components and extent to which they use intervention resources (i.e. exposure or dose received), and 4) describe the setting (i.e. contextual factors, facilitators, barriers, contamination) into which the intervention is being implemented that may have an influence on intervention effectiveness [53, 54]. Detailed process evaluation objectives, data sources and indicators are presented in Table 3. Process evaluation data will be collected two months after baseline (when infants are 8 months-old) and end of intervention (when infants are 15 months-old). Structured and semi-structured interviews, review of records (e.g. training and education attendance sheet, activity logs, training test scores, etc.) and structured observations will be carried out with intervention participants (mothers, nutrition counsellors, health workers).

**Table 3:** **Process evaluation objectives, data collection methods and indicators**

Objectives and data sources	Description of process indicators	Characteristic of process indicator
**Assess whether the intervention activities are implemented as planned**		
Activity logs	Number of taught modules and sessions held with nutrition counsellors	Fidelity
	Number of education and counselling sessions and taught lessons held with mothers	
	Number of reading resources distributed to targeted groups	
	Number of home visits conducted by nutrition counsellors	
	Number of sensitisation meetings held with family members	
	Number of sensitisation meetings held with health facility staff	
Supervisory reports	Review of counsellors’ workbooks for completeness, validity of documented information, referrals, appointments kept or missed	Fidelity and dose delivered
Registration forms	Number of community-based nutrition counsellors trained	Fidelity
	Number of health facility staff sensitised	
Pre- and post-test scores	Performance of nutrition counsellors in knowledge and skills gained during training	Fidelity and dose delivered
Evaluation forms	Quality of training sessions (adequacy of delivery methods, time allocated to sessions, usefulness of materials and field practice, attitude to training)	
Structured observations	Nutrition counsellor’s skills during home visits on interpersonal skills, use of reading resources, problem-solving, confidence in counselling mothers	
**Evaluate the extent to which the intervention reaches the intended mothers and families**		
Activity logs	Number of recruited infants in intervention and control villages. Number dropped out. Reasons for dropping out	Reach (participation rate)
Attendance records	Number of mothers attending each education and counselling session (plus cooking demonstration)	
	Number of family members represented in sensitisation meetings	
	Number of mothers visited/attended during home visits	
**Determine the degree to which targeted mothers are exposed to intervention components and extent to which they use intervention resources**		
Attendance records	Number of mothers attending each education session	Dose received (exposure)
	Number of mothers with information booklets	
Structured observations	Observation of mothers’ attentiveness, interest/keen (e.g. asked/answered questions, give examples) during education and counselling sessions	
	Observation of mothers’ feeding style (e.g. responsive feeding) during cooking demonstration sessions	
	Amount of meal consumed by infants at cooking demonstration sessions	
Semi-structured interviews	Number of mothers who could recall (spontaneously and/or prompted) key behaviours learned in education sessions and home visits	
	Number of mothers who could recall (spontaneously and/or prompted) messages contained in information booklet	
Semi-structured interviews	Mothers’ perception on usefulness and preference (by ranking) of intervention aspects (education sessions, sensitisation meetings, home visits, booklet).	Dose received (satisfaction)
	Mothers’ level of satisfaction with counsellors’ services	
**Investigate setting into which intervention is being implemented that may influence intervention effectiveness**		
Semi-structured interviews	Interviews with nutrition counsellors about any ongoing interventions (e.g. competing programmes), perception regarding intervention delivery, strengths, challenges, and suggestions for improvement	Context, facilitators, barriers, contamination
	Interviews with village and sub-village leaders about village profile during baseline	
Structured interviews	Interviews with residence in-charge of health centre on presence (and number) or absence of health staff who attended sensitisation meetings	
Structured observations	Observation of health facility staff conducting health education sessions at the facility in intervention and control villages	

A process evaluation will also be conducted in control villages when infants are aged 8 and 15 months-old to identify and describe actions, events and context which may reveal new interventions, evidence of contamination or other factors external to the intervention. The information will be collected from village leaders and health workers through structured interviews and observations.

### Monitoring of intervention

Data on the primary and secondary outcomes of the study will be collected through periodic monitoring and at the end of study. A list of intervention activities and their schedules is developed to facilitate the development of a monitoring plan for assessment of intervention progress. The activity monitoring plan will document delivery of intervention activities on weekly and monthly basis. The research team will visit intervention villages every 2 weeks in the first 2 months of intervention, and thereafter visits will be made at 2-months interval. Researchers will log and report all activities related to the delivery and quality of training, education, supervision and sensitisation meetings using monitoring tools (e.g. forms, logs, observation checklists, supervisory reports). Nutrition counsellors will document home visits in workbooks. Actions or events external to the intervention which happen in the intervention villages will be documented in event forms and semi-structured interviews.

### Trial status and schedule of intervention activities

The trial is planned to begin in September 2014 with the training of community-based nutrition counsellors. This will be followed by recruitment of infants and parents, collection of baseline information, education and counselling session, sensitisation meetings and home visits. In between, supervision, monitoring and data collection will be conducted. A summary of activities and measurements planned during the intervention period are presented in Table 4.

**Table 4:** **Schedule of intervention activities and measurements during the study period**

Activities and measurements	Child’s age (in months) and time points of data collection									
	6 ^a^	7	8	9 ^b^	10	11	12 ^c^	13	14	15 ^d^
**Activities in Intervention group**										
Train community-based nutrition counsellors	X						X			
Education and counselling of mothers	X			X			X			
Sensitisation meetings	X						X			
Home visits by counsellors	X	X	X	X	X	X	X	X	X	X
Supervision of community-based nutrition counsellors	X	X			X			X		
Monitoring	X	X			X			X		
**Measurements**										
Covariates	X^I+C^									
Child anthropometry	X^I+C^			X^I^			X^I+C^			X^I+C^
Child feeding practices (frequency, diversity)	X^I+C^			X^I^			X^I+C^			X^I+C^
Child food intake (24-hr recall)	X^I+C^						X^I+C^			
Child morbidity & healthcare-seeking	X^I+C^			X^I^			X^I+C^			X^I^
Child haemoglobin concentration	X^I+C^						X^I+C^			
Maternal recall and practice of feeding recommendations				X^I^			X^I^			X^I^
Process evaluation data			X^I+C^							X^I+C^

^**a**^Baseline. ^**b**^Follow-up 1. ^**c**^Follow-up 2. ^**d**^End of intervention. X^I+C^ Measurements taken in Intervention and Control groups; X^I^ Measurements taken in Intervention group alone.

### Data management and analysis

Data will be entered in Epi-data version 3.1 and consistency analysed by range checks of data values. Data will be analysed using STATA release 12.0 (STATA Corporation, Texas, 2007). Frequency distributions will be run to identify outliers. Impact evaluation data will be compared between intervention and control villages using hierarchical or multi-level models and presented at the cluster and individual levels. The methods will provide adjustment for potential covariates and confounders at the cluster and individual levels. Data will be analysed by intention to treat. Linear mixed models will be used for continuous outcomes (e.g. growth parameters) with cluster, household, and child as random effects to account for clustered observations. Generalised linear mixed models will be used for discrete outcomes (e.g. frequency of specific food intakes, practices, etc.). Random effects logistic regression analyses, taking clustering into account, will be used where appropriate. Values of p < 0.05 will be considered statistically significant.

The process evaluation data will use both inductive and deductive approaches [55] to analyse qualitative data. The data will include structured observation checklists, in-depth interviews, and documents. From these, frequent, major, or significant themes inherent in raw data will be identified and coded. After coding, the transcripts will be entered in and analysed using NVivo version 10 (QSR International, Australia). Content will be analysed in terms of countable concepts/objects e.g. descriptive statistics, or in the broadest terms (e.g. majority, some, very few). Direct quotations from the group discussions will also be provided. Key analytical categories will be identified and interpreted to explore all aspects of intervention delivery, identify factors contributing to intervention success or failure, if intervention results have been affected by implementation process, and document encountered barriers. Within the intervention group, outcomes will be compared between groups of people with high and low scores for a process evaluation characteristic.

## Discussion

By offering age-specific, practical problem-solving education and counselling on feeding and health care practices, and ensuring continuous support, it is expected that the nutrition education package will result in a larger impact on dietary adequacy, growth and health than could be achieved through routine health education alone. The package will look into improving the quality of nutrition education, interpersonal skills needed to communicate nutrition recommendations and capacity of families to act on nutrition recommendations. The package will achieve these through the implementation of its components: education and counselling mothers; training community-based nutrition counsellors and monthly home visits; sensitisation meetings with families and health facility staff; and supervision of community-based nutrition counsellors. With respect to the multi-component nature of this intervention, understanding of outcome results and implementation pathway need to be well documented. Implementation or process data are critical to understand what, how and why an intervention works or does not work. For a successful intervention to be replicated, data on implementation are necessary [54]. In developing country settings, where resources are limited and basic public health problems prevalent, knowledge of implementation is critical to promote effective programmes, their replication and expansion [56].

This intervention study is in line with a global focus on infant and young child nutrition. In addition, infant and young child feeding is one of the priority areas specified in the Tanzania’s National Nutrition Strategy (July 2011-June 2016). The intervention has included components that are likely to enhance maintenance of behaviour after completion of intervention. Mothers, caregivers and family members who participate in the intervention will serve as future reference points for other households. To influence long-lasting behaviour change, trained community-based nutrition counsellors will remain a valuable resource to their respective villages and district. Sensitised health facility staff will apply gained knowledge and attitudinal change to counsel mothers during routine well-baby and sick-child visits. The intervention components can feasibly be integrated into existing health services and district health plans. If successful, the approach used to deliver the nutrition education package could be easily and inexpensively scaled and disseminated in other rural villages in the country.

This study has some limitations. First of all, potential risks may arise due to lack of double blinding. Parents of study infants and nutrition counsellors allocated to intervention group may be aware of the allocated arm. To minimise this limitation, data collectors will be kept blinded to village allocation and will not be residents in intervention or control villages. Intervention implementers will not be part of the team collecting outcome data. The second limitation is that self-reporting measurements such as maternal recall of recommended practices may overestimate nutrition outcomes and behaviours due to recall bias. In this trial, the recall period will be relatively short (past week or past two weeks) to optimise the reliability of self-reports. Thirdly, the intervention duration of 9 months is relatively short to assess change of certain behaviour and the sustained effects. However, it was observed in previous trials that similar outcome measures demonstrated change within 6 months of intervention [15], and the effects were sustained 3 and 12 months after intervention [57]. This trial is designed to assess outcomes within the common intervention duration of 6 months as well as follow-up measurements to allow evaluation of short and mid-term effects.

## Electronic supplementary material

Additional file 1: **A conceptual framework illustrating intervention components and pathways among targeted behaviours and mediating factors for improvement of child growth.** A conceptual framework. (DOCX 15 KB)

Additional file 2: **Matrix of intervention objectives, behavioural performance objectives and determinants: application of the theory of planned behaviour.** Matrix of intervention objectives, behavioural performance objectives and determinants. (DOCX 16 KB)
