# Supplementary material for: Effectiveness of a nutrition education package in improving feeding practices, dietary adequacy and growth of infants and young children in rural Tanzania: rationale, design and methods of a cluster randomised trial
Source: BMC Public Health. 2014 Oct 16;14:1077. doi: 10.1186/1471-2458-14-1077 (PMC4216379; doi:10.1186/1471-2458-14-1077)
Supplement: Supplementary file 1 — Additional file 1: A conceptual framework illustrating intervention components and pathways among targeted behaviours and mediating factors for improvement of child growth. A conceptual framework. (DOCX 15 KB) [file 12889_2014_7188_MOESM1_ESM.docx]

# Additional file 1. Conceptual framework illustrating intervention components and pathways among targeted behaviours and mediating factors for improvement of child growth

Increased nutrients intake

Improved growth

Improved health status

Breast feeding beyond 1 year

Maternal behaviour (feeding, hygiene, health)

Maternal intentions & decision (plan/expect to apply recommendation)

Maternal social influences (social encouragement, pressure from family & friends)

Early detection of illness, healthcare seeking, disease prevention

Home meals (variety, frequency, amount, safety, responsive feeding)

Maternal attitudes toward behaviour

Maternal perceived behaviour control or self-efficacy (beliefs on ability to perform behaviour)

Community-based nutrition counsellors

Mothers & Caregivers
