# Supplementary material for: Effectiveness of a nutrition education package in improving feeding practices, dietary adequacy and growth of infants and young children in rural Tanzania: rationale, design and methods of a cluster randomised trial
Source: BMC Public Health. 2014 Oct 16;14:1077. doi: 10.1186/1471-2458-14-1077 (PMC4216379; doi:10.1186/1471-2458-14-1077)
Supplement: Supplementary file 2 — Additional file 2: Matrix of intervention objectives, behavioural performance objectives and determinants: application of the theory of planned behaviour. Matrix of intervention objectives, behavioural performance objectives and determinants. (DOCX 16 KB) [file 12889_2014_7188_MOESM2_ESM.docx]

# Additional file 2. Matrix of intervention objectives, behavioural performance objectives and determinants: application of the theory of planned behaviour

| **Intervention objectives (IO) and behavioural objectives (BO)** | **Performance objectives (target behaviours)** | **Determinants** |
| --- | --- | --- |
| **IO 1. To improve feeding practices** |  | **Personal:**  KS.1. Describes how child feeding practices and child health principles impacts on child growth and overall health  KS.2. Demonstrate how to provide nutritious meals and feed her child responsively  AT.1. Believes that appropriate feeding and health practices is important to ensure optimal growth and is achievable  AT.2. Expresses positive attitude towards nutritional benefits of adopting optimal child nutrition practices  AT.3. Believes in obtaining support from family members  PBC.1. Express confidence in understanding optimal feeding practices  PBC.2. Express confidence in recognising the need for appropriate treatment of illnesses  PBC.3. Express confidence in own ability to try the recommended practices  **External:**  SN.1. Perceives family, friends, peers, and community are expecting them to practice recommended behaviours  SN.2. Recognise that mothers with children of similar age in the village are also putting into practice the recommended behaviours  SN.3. Receives support/reinforcement from nutrition counsellors and health facility staff |
| BO 1.1. Increase awareness & knowledge on feeding | Continue to breastfeed as often as the child wants throughout the first and second years |  |
|  | Introduce other fluids and foods to your child at 6 months |  |
|  | Gradually increase meal consistency, variety, amount as child gets older |  |
| BO 1.2. Increase knowledge on benefits of different varieties of foods | Identify & group locally available foods for making typical meals |  |
|  | Select nutrient-dense foods |  |
|  | Prepare nutritious meals |  |
| BO 1.3. Increase feeding during child illness & recovery | Encourage your child to eat with patience and love |  |
|  | Offer more frequently fluids, soft, varied, and appetizing foods/meals during illness & recovery |  |
|  | Increase frequency of breastfeeding during illness & recovery |  |
| BO 1.4. Handle foods and meals in hygienic way and prepare safe meals | Wash hands before handling food & during meal preparation |  |
|  | Wash hands after using the toilet or cleaning a child |  |
|  | Keep your home & compound clean |  |
| BO 1.5.Increase utilisation of health services during illness | Identify danger signs of childhood illnesses |  |
|  | Seek health care promptly at health facilities |  |
| BO 1.6.Adhere to medical treatment | Comply to treatment & advice |  |
|  | Protect children from communicable diseases |  |
| **IO 2. To improve dietary adequacy** |  |  |
| BO 2.1. Increase diversity in diets | Add legumes in each meal |  |
|  | Feed eggs, beef, pork, chicken, liver, fish, or sardines at least 3 times per week |  |
|  | Add vegetables in each meal |  |
|  | Give a fruit at least once per day |  |
| BO 2.2. Increase frequency | Increase number of meals per day |  |
|  | Increase number of snacks per day |  |
| BO 2.3. Increase nutrient density | Prepare thick porridge made from a combination of foods |  |
|  | Add milk to porridge |  |
|  | Increase amount & consistency |  |
| BO 2.4. Maintain breastfeeding | Continue to breastfeed as often as the child wants throughout the first and second years |  |
|  | Breastfeed first before giving other meals |  |
| **IO 3. To improve growth** |  |  |
| BO 3.1. Reduce stunting | Perform optimal feeding practices |  |
| BO 3.2. Reduce underweight |  |  |
| BO 3.3. Reduce prevalence of acute respiratory illnesses and diarrhoea | Ensure prompt and appropriate treatment during illness |  |

KS=Knowledge and Skills; AT=Attitude; PBC=Perceived behavioural control; SN=Subjective norms
